# Unravelling the variations of the society of England and Wales through diffusion mapping analysis of census 2011

**DOI:** 10.1098/rsif.2023.0081

**Published:** 2023-08-23

**Authors:** Gezhi Xiu, Huanfa Chen

**Affiliations:** ^1^ School of Earth and Space Sciences, Peking University, Beijing, People’s Republic of China; ^2^ Centre for Complexity Sciences and Department of Mathematics, Imperial College London, London, UK; ^3^ Centre for Advanced Spatial Analysis (CASA), University College London, London, UK

**Keywords:** census, manifold learning, localization, urban sciences

## Abstract

We propose a new approach to identify geographical clustering and inequality hotspots from decadal census data, with a particular emphasis on the method itself. Our method uses diffusion mapping to study the 181 408 output areas in England and Wales (EW), which enables us to decompose the census data’s EW-specific feature structures. We further introduce a localization metric, inspired by statistical physics, to reveal the significance of minority groups in London. Our findings can be adapted to analogous datasets, illuminating spatial patterns and differentiating within datasets, especially when meaning factors for determining the datasets’ structure are scarce and spatially heterogeneous. This approach enhances our ability to describe and explore patterns of social deprivation and segregation across the country, thereby contributing to the development of targeted policies. We also underscore the method’s intrinsic objectivity, guaranteeing its ability to offer comprehensive and unbiased analysis, unswayed by preconceived hypotheses or subjective interpretations of data patterns.

## 1. Introduction

Understanding the demographic and socio-economic characteristics that shape society is critical. However, extracting meaningful insights from scale-relevant and spatially heterogeneous collections of social, and potentially biological or physical variables, is challenging. The distribution of social classes and groups significantly impacts regional stability, social welfare and economic potential [1–3]. For example, [4] shows in some cities, the number of university students and social deprivation are paramount in explaining other social variables in census statistics. Other research illuminates the impact of social, economic and ethnic attributes on regional disparities, such as energy burdens in households [5], heterogeneity in epidemic vulnerability [6] and environmental inequality [7].

Census data, or similar datasets, often group a large number of social variables collected from small areas across an entire country into spatial distributions of a few independent composite indices [8]. Nevertheless, due to the scale and complexity of these datasets, there are practical challenges. These include the selective processing of social variables on a large spatial scale or using a full collection of social variables for only a small region [9].

We posit that deriving these social variables or spatial regions from census data can compromise objectivity. For example, defining geographical clustering of social groups for statistical analysis requires aggregating regions into specific areas. However, the modifiable areal unit problem (MAUP) [10–13] challenges the possibility of such spatial aggregations being consistent across different social issues. Also, heterogeneity of the social variables across cities creates a gap between local studies and the general significance of these features. Furthermore, researchers’ differing perspectives on nominal attributes like race or religion can lead to a lack of consensus on these features’ significance. These issues make synthesizing findings from different studies to identify critical socio-economic characteristics challenging [10].

To effectively analyse census data and overcome the inherent challenges, the diffusion maps (DMs) manifold learning method has proven to be a valuable tool. This method captures the interplay of social indicators by representing the social identities of different locations as branches in the data manifold. Previous studies, such as [4], have demonstrated the effectiveness of DMs in dissecting and identifying key social indicators within similar cities, successfully transforming complex, high-dimensional census data into more understandable components, as evidenced in the cases of Bristol and Edinburgh.

However, while successful in these applications, it remains crucial to ensure these methodologies are comprehensive and accurately represent the entire population of England and Wales (EW). Recognizing the need to identify globally consistent contributors while also respecting the unique characteristics of small communities, our study applies DMs to the complete census data of EW. The goal is to uncover geographical clustering and inequality hotspots, thereby ensuring a more detailed understanding of the nation’s demographics. By combining the capacity to analyse large-scale patterns with the ability to identify localized specifics, our approach provides a nuanced and accurate depiction of demographic patterns.

In this work, we employ a two-stage process: initially, we form clusters of observed values for multiple sociodemographic variables based on their similarities; subsequently, we scrutinize these clusters for discernible local patterns, thus addressing both traditional clustering and the identification of local ‘hotspots’ as described in spatial analysis literature [14,15]. Our study uses DMs to analyse the census data of EW and identify geographical clustering and hotspots of inequality. The goal is to decompose high-dimensional social variables into branched, interdependent social factors, revealing patterns in space that would otherwise go unrecognized. A new method, the correlation table, is proposed to explain the derived social dynamics and provide a standard for structuring and analysing any spatial collection of features while minimizing pre-assumed spatial correlations in large study areas, such as a densely organized country. Additionally, we introduce a localization metric to reveal the critical features of specific cities. Our method provides a comprehensive view of the descendingly important features of EW and tracks where these features are locally highlighted. By combining the DMs method with the correlation table and localization metric, our study offers a powerful set of tools for understanding demographics and uncovering patterns in social and economic data.

## 2. Method and data

### 2.1. The census data

The 2011 UK Census, conducted by the Office for National Statistics of the United Kingdom (https://www.ons.gov.uk/census), presents a thorough delineation of the population and households in EW. This rich dataset, consisting of over 1000 social variables or features, is organized across 181 408 compact, locally homogeneous output areas (OAs). These OAs, designed to encompass between 125 and 650 households, represent the smallest geographical units used in the census. The area of an OA can vary significantly: In the London area, the average size of an OA is approximately 0.0655 km^2^, derived from dividing the city’s total area of 1572 km^2^ by its approximately 24 000 OAs; across EW, the size of an OA would similarly be determined by the total land area of approximately 151 174 km^2^ and the total number of OAs (181 408). This gives a rough average size of an OA as approximately 0.83 km^2^. Despite the wealth of information encapsulated within this data, the sheer volume can hinder the extraction of meaningful insights. Our research navigates this challenge by employing DMs. This methodology allows for a decomposition of the high-dimensional social variables into branched social factors, unveiling concealed spatial patterns and intricate relationships among the local values of these factors.

### 2.2. Diffusion maps

Diffusion mapping is a nonlinear dimensionality reduction technique that leverages a random walk process on a sparse network of data points to uncover the structural differentiation within data. In urban sciences, it is a sensible approach based on the idea that locations are clusters of similar individuals. Hence, the similarity of locations is strongly associated with their distances to each other in the data space, which can further be used to define the network topology. This method allows for a local perspective to be integrated into a broader understanding of urban dynamics, making it an ideal tool for our study.

The constructions of the DM are performed as follows. Suppose for each of the *N* OAs, *x* is a *M*-dimensional vector whose entries are the social variables. Here, *M* is the dimensionality of social variables in the census dataset, and *N* is the total number of data points. To leverage the distribution heterogeneity of different social variables, we measure the similarity *s*(*x*, *y*) of the OA pair *x*, *y* through their Spearman rank correlation. Notably, this correlation is not based directly on geographical proximity but on the ranks of the *M*-dimensional vectors of social variables.

$$\rho_{x,y}=\frac{R_{x}R_{y}-N^{2}/4}{\left\|R_{x}-n/2\|\cdot \|R_{y}-n/2\right\|},$$

for each pair (*x*, *y*) in {1, …, *N*}, where *R*_*x*_ is a vector that each of its entries is the rank of *x* for a social variable. We denote Σ as the rank correlation matrix, where each of its elements $\Sigma_{x,y}=\rho_{x,y}$ is the correlation of the corresponding data points *x* and *y*. The elements of Σ are thus all valued between −1 and 1. Nearby points in the data space have *ρ*_*x*,*y*_ close to 1 following a framework in [16]. To emphasize the structure of the most important links connecting most similar data points, we define an alternative matrix $\tilde{W}$ keeping only *k* largest elements in each row of Σ and set the rest of elements be zero. Here, we choose *k* = 10 that barely keeps the network connected; that is, from each data point there exists at least one route to every other data point in the network. Next, we define a *N* × *N* normalization matrix *D* whose diagonal elements are the row sums of Σ. Then, we compute the eigenvalues and right eigenvectors of the following normalized Laplacian matrix $A=I-D^{-1}\tilde{W}$. *A* can be regarded as a Markovian transition matrix for a random walk process over data points. The random walk process converges to a continuous time diffusion process as *N* → ∞ and a small *k* over the observable data manifold. The low-order eigenvectors of *A* are then an approximate parametrization of the underlying manifold that hints at the actual urban dynamics.

Our choice to use rank correlation over direct correlation brings several advantages. It provides a robust measure of association that minimizes the impact of extreme values and imbalanced distributions, generating a comparable data space. The rank of locations in certain social variables determines their linkage, enabling the model to handle both vertical (among different variables) and horizontal (across different locations) dimensions. This sets our approach apart from more conventional clustering methods such as *k*-means, principal components analysis (PCA) and factor analysis. Unlike *k*-means, our method incorporates both vertical and horizontal dimensions. PCA offers a global metric and is not equipped to handle geographical heterogeneity, while factor analysis, when faced with the high dimensionality of our data space, becomes impractical due to its need for a predefined target.

As presented in [4], the social features can be represented by the linear combination of the leading eigenvectors. The complete set of eigenvectors *η*_*j*_ correspond to an increasing sequence of *A*’s eigenvalues $\lambda_{0}\leq \lambda_{1}\leq \cdots \leq \lambda_{N-1}$, and each of *η*_*j*_ corresponds to a relatively independent dynamical variable, whose nonlinear combinations are explicit in the census as social variables. We then colour-code the OAs according to their corresponding elements in each eigenvector *η*_*j*_, and generate maps to visualize the spatial configurations of the dynamical variables.

In order to make sense of the dynamical variables identified through the eigenvectors, we perform a backward calculation to investigate the correlation between the eigenvectors *η*_*j*_ and the census social variables. By identifying the social variables that are most positively and negatively correlated with a given eigenvector *η*_*j*_, we can gain valuable insights into the significance of the corresponding eigenvectors. This information, in combination with the visual representation of the eigenvectors through their spatial plots (i.e. maps), enables a comprehensive analysis of the underlying dynamics.

### 2.3. Virtual similarity networks versus social hierarchy

In our use of DM, we frame the census dataset as a weighted sparse network composed of 181 408 OAs in a 1450-dimensional space, with the ‘similarity’ of OAs acting as the weights of the connections. The sparsity of this census data network is needed to uncover the central structure of the feature’s synthetic structure, rather than being dominated by a few highly heterogeneously distributed social variables. The next logical question becomes how sparse the network should be to recover the inherent structures and properties of the census data, such as social hierarchy and criticality [17]. Therefore, our discussion on whether there are significant cross-scale features in the census data helps to validate our proposed network, which is grounded in local metrics.

We approach the sparsity problem by making the assumption that the census data network should exist in a state of most informative criticality. The notion of ‘criticality’ comes from the widely accepted view of society as a complex system that operates in a state of balance between order and disorder, or stability and instability, much like the concept of criticality in physics and ecology [18].

As the census data network is formed by finding the *k*-most similar OAs (in terms of census statistics) for each of the OAs, the sparsity of the network can then be determined through the value of *k*: a larger (smaller) *k* represents stronger (weaker) network connectivity, and denote *G*_*k*_ as the network of connectivity *k*.

To define the census data network’s criticality, we specifically consider the degree distribution of the census data network for different network sparsity [19]. Table 1 shows that as the similarity network *G*_*k*_ is defined as more sparse (i.e. smaller *k*), the likelihood of *G*_*k*_’s degree distribution being more similar to a power-law distribution increases. This suggests that as the adjacency threshold and network connectivity decrease, the network’s power-law characteristics become more prominent. The analysis in this paper thus chooses *k* = 10 to maximize the likelihood for the data network to be power-law-like.

**Table 1.** The maximum-likelihood fitting methods combined with goodness-of-fit tests (based on the Kolmogorov–Smirnov statistic and likelihood ratios) of *G*_*k*_’s degree distribution. Columns *α* and *x*_min_ represent the estimation of the power-law degree distribution of the census network of OAs with the form $p(k)=k^{-\alpha }/\sum_{n=1}^{\infty }{\left(n+x_{\min}\right)}^{-\alpha }$, where *x*_min_ is the minimum degree, and *α* is the power-law exponent. *R* is the likelihood ratio test comparing the fit of the power laws curve and the log normal curve. A more positive *R* indicates a better fit of the power-law curve to the degree distribution over the log normal curve. Finally, the *p* column is the *p*-value of the confidence of the power-law distribution.

*k*	*α*	*x*_min_	*R*	*p*-value
10	2.890	16	0.933	1.846 × 10^−9^
20	2.877	30	0.0385	9.367 × 10^−1^
30	2.878	44	−2.726	1.381 × 10^−1^
40	2.875	58	−9.835	4.938 × 10^−3^
50	2.867	73	−27.09	3.493 × 10^0^

### 2.4. Localized inverse participation ratio

The diffusion mapping eigenvectors are globally consistent features of significant importance in the distribution of various social variables found in census data. Dominant factors undoubtedly contribute to the society of EW, but less dominant ones can also have regional significance. As in the example of Bristol and Edinburgh, the number of university students may carry higher socio-economic weight than social deprivation. However, the spatial distribution of the eigenvectors, as depicted by *η*_3_ (the prison establishments, elaborated in the following section), are discontinuous because the similarity of OAs is defined through the ranks of census social variables. Consequently, traditional hotspot detection methods relying on spatial autocorrelations may fail to reveal the localized importance of these eigenvectors in specific cities.

In this context, we highlight the importance of understanding how globally significant factors, represented by leading DM eigenvectors, localize within specific cities. For this purpose, we introduce a new metric, the local inverse participation ratio (LIPR), enabling us to trace the localization of an eigenfeature (a specific factor encapsulated by an eigenvector) into a given city. We argue this is crucial as it aids in identifying cities with special importance for certain factors. While numerous metrics for analysing local spatial properties exist, including Anselin’s local indicators of spatial association [20], Lloyd’s local models for spatial analysis [21], Local Moran I and Getis–Ord indices [22,23], few methodologies are available that specifically measure the local and spatially discontinuous characteristics of global features. We plot the maps of Getis–Ord Index and Local Moran I of the top 20 DM eigenvectors in the electronic supplementary material [26], from which identifying significant patterns revealing socio-economic properties is difficult. Hence, the necessity of introducing a new localization index such as LIPR.

The LIPR is an extension from the metric *inverse participation ratio* (IPR) from statistical physics [24], defined as

2.1 $${\mathrm{IPR}}_{i}=\sum_{k=1}^{N}\frac{{\left(\eta_{i}^{k}\right)}^{4}}{\sum_{\;j=1}^{N}{\left(\eta_{i}^{\;j}\right)}^{2}},$$

where *N* is the number of *η*_*i*_’s entries thus the number of OAs, and $\eta_{i}={\left(\eta_{i}^{1},\;\ldots ,\;\eta_{i}^{N}\right)}^{T}$. Here, if a feature appears in one single area, i.e. *η*_*i*_ = (0, …, 0, 1, 0, …, 0)^*T*^, the corresponding IPR_*i*_ = 1; for another limiting case, if a feature is uniformly distributed in all the areas, $\eta_{i}={\left(1/\sqrt{N},\ldots ,1/\sqrt{N}\right)}^{T}$, the corresponding IPR_*i*_ = 1/*N*, which diminishes as *N* grows. So a highly localized pattern corresponds with a large value of the IPR. The LIPR does not inherently incorporate geographical proximity, in the traditional sense, into its calculation. Instead, it emphasizes the intensity of an eigenfeature in a certain area. Therefore, two regions receiving high weights, even if far apart geographically, may indeed have the same LIPR score as two geographically adjacent areas with similar high weights. This potentially allows for the identification of areas of significance for a particular factor, irrespective of their geographical distribution. Building on the distinction between *detection of clusters* and *detection of clustering* that is made in [14], the LIPR metric, in this context, leans more toward *detection of clustering*. It suggests *locality* in terms of eigenfeature intensity, rather than geographical proximity.

To capture whether an indicator clusters in an area, we extend the IPR to LIPR of area *X*,

2.2 $${\mathrm{LIPR}}_{i}^{X}=\left(\frac{\sum_{\;j\in X}{\left(\eta_{i}^{\;j}\right)}^{4}}{\|\eta_{i}{\|}^{2}}\right)/\left(\frac{\sum_{\;j}{\left(\eta_{i}^{\;j}\right)}^{4}}{\|\eta_{i}{\|}^{2}}\right)=\frac{\sum_{\;j\in A}{\left(\eta_{i}^{\;j}\right)}^{4}}{\sum_{\;j}{\left(\eta_{i}^{\;j}\right)}^{4}}.$$

It is intended to be large when the distribution of eigenvector *i* is highlighted in the city *A*. A region with a high LIPR indicates the spatial clustering of small communities, which supports similar social groups across the country, and is mainly localized in some individual cities.

The LIPR metric can be used to understand how localized an eigenvector is in a certain city. We give two examples to illustrate how the metric works in two limiting cases. In the first case, an eigenvector highlights only one area in London and assigns it a value of 0.1, while assigning 0 to all other areas. The corresponding LIPR in this case would be a relatively high value of 0.001. In the second case, if an eigenvector does not highlight any specific areas in London and assigns all 10 000 areas a value of 0.0001, the corresponding LIPR would be a near-zero value of 10^−8^. We explain that in general, a highly localized eigenvector would have a larger LIPR value and that the metric can be used to pinpoint meaningful communities in more than one city.

## 3. General dominant features

We begin at the smallest positive, thus the most important Laplacian eigenvectors of the EW diffusion mapping. A map can associate each of the eigenvectors, which is colour-coded from the most negative to the most positive entries, representing the exposures of each OA to the corresponding demographic context.

### 3.1. Urbanization properties

**Figure 1.**
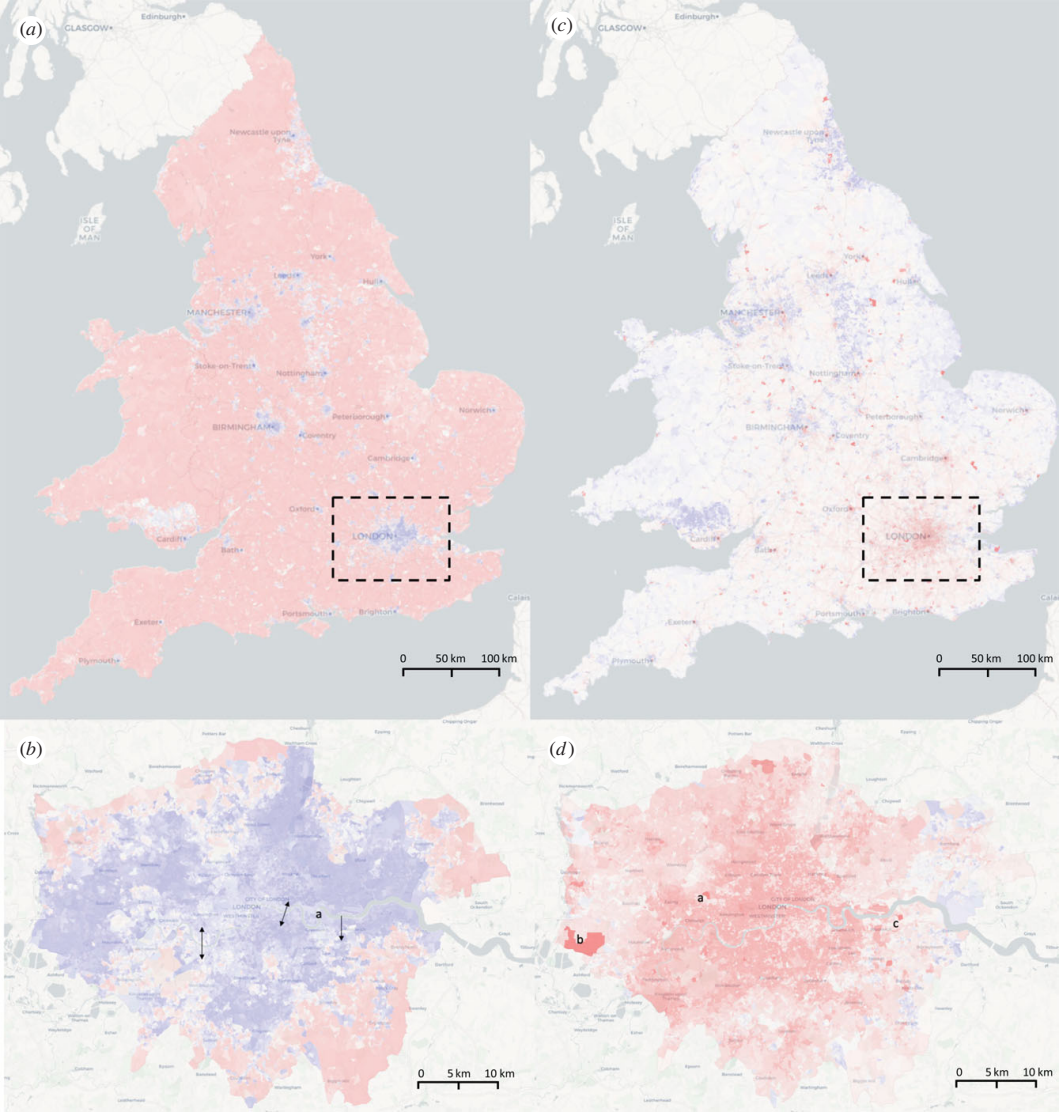
The eigenvector map of *η*_1_ and *η*_2_, the representative eigenvectors, that shows global properties. The colours are assigned by the entries of an eigenvector, from the most positive (red) to the most negative (blue). Here, *η*_1_ highlights the main cities; *η*_2_ pinpoints the most skilled workers, which are mostly concentrated in the main airports. The label a in (*b*) is the Blackwell tunnel from where the symmetric pattern is broken between either sides of Thames; In (*d*), a, b and c are Hammersmith Hospital, Heathrow Airport and HM Prison Isis, respectively.

The first eigenvector, *η*_1_ can be used to identify patterns of urbanization in EW (figure 1*a*,*b*). *η*_1_’s negative values are localized in the main cities of the country, and it highlights not only the largest cities such as London, Liverpool and Manchester, but also smaller central places surrounded by forest and mountains in the form of a continuous patch of OAs represented by Porthmadog, Tregaron and Newport. By analysing only London entries of *η*_1_, we find working-class residential areas expanding along the River Thames, with a relatively north–south symmetrical pattern from west to east until the Blackwall tunnel neighbourhood, where tunnels replace the walkable bridges as the connection between the riversides. We conclude that residential urban area is continuously defined as walkable neighbourhood, which is the most explanatory feature of the 2011 census. We recall the diffusion mapping results inputting the city-level census data in [4] that highlight universities and poverty as the dominant features of Bristol and Edinburgh. The eigenvector *η*_1_ exhibits a more globalized spatial distribution of urbanization.

A natural question to follow is what element from the census perspective determines the shape of a city identified by *η*_1_. To this end, we compute the correlation of *η*_1_ with all the census social variables. We find that the most negatively correlated census variables of *η*_1_ on the distribution of all OAs in EW (and the corresponding correlations) are: *living in a couple: Married or in a registered same-sex civil partnership* (−0.82), *two cars or vans in household* (−0.80), *Married* (−0.78), *Occupancy rating (rooms) of +2 or more*, i.e. at least 2 rooms more than the basic standard (−0.78); Meanwhile, *η*_1_ is also highly correlated with particular races and religions (*Black African/Caribbean/Black British: African* −0.58, *Muslim* −0.57). These social variables capture the typical community in a city in EW. We note that urbanization is the most important dimension in census, and urbanization is largely explained by the percentage of marriage and civil partnerships, vehicles ownership and occupancy status of the households in a neighbourhood.

Eigenvector *η*_2_ highlights similar areas as those identified by *η*_1_ but exhibits a milder aggregation with many clustered areas in medium-level regional centres (figure 1*c*,*d*). Generally, *η*_2_ picks all the important airports in EW with the highest entries, in addition to a general mapping of the working class in most cities and lower-level central places. We conclude that *η*_2_ is mostly associated with the skilled occupations, which can also be validated statistically by its most correlated census variables of degrees and diplomas: *Degree (for example BA, BSc), Higher degree (for example MA, PhD, PGCE)*, 0.85, *two+ A levels/VCEs, 4+ AS levels, Higher School Certificate, Progression/Advanced Diploma, Welsh Baccalaureate Advanced Diploma* (0.81), *Highest level of qualification: Level 4 qualifications and above* (0.81). *η*_2_’s high correlation with education and its appearance at the second most dominant eigenvector indicate that education is one of the most clustering feature of EW, that widely explains other socio-economic properties underlying census data.

We then wonder what areas are ‘most educated’. Zooming in on London, *η*_2_ separates the city from Northwestern to Southeast, similar to what is usually believed as the separation of Old and New London. The most highlighted areas of *η*_2_ in London are the Hammersmith Hospital. However, *η*_2_ surprisingly finds HM Isis Prison. We referred to the prison website and Wikipedia and learned that this prison provides education and vocational training in partnership with Kensington and Chelsea College.

Beyond educations, *η*_2_ is highly negatively correlated with *Routine occupations* (−0.75), *No British identity* (−0.60) and *Bad health* (−0.59). These features indicate that education is one of the most important determinations of household gathering features as the education-related eigenvector appears to be as *η*_2_. Here, we compare the spatial distribution of *η*_2_ and *η*_6_ because visually *η*_6_ finds almost every university in EW. We conclude that *η*_2_ is more about where the university graduates settle and work, while the positive entries of *η*_6_ find most of the university campuses. The population composition of *η*_6_’s most correlated with the racial census variables are *White: English/Welsh/Scottish/Northern Irish/British* (0.57), *No religion* (0.55) and *Born in UK* (0.53). These features can be linked to the typical features of the university neighbourhood of EW.

### 3.2. University neighbourhoods

**Figure 2.**
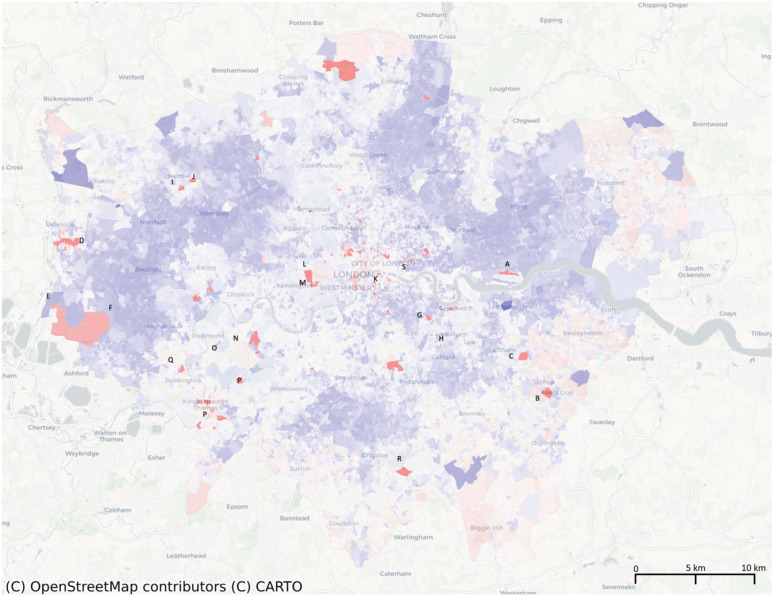
The eigenvector *η*_6_ zoomed in London, which finds A, University of east London; B, Queen Mary’s Hospital; C, University of Greenwich; D, Brunel University; E, Smart College UK; F, The London College; G, Goldsmiths, University of London; H, Lewisham College; I, Harrow School; J, Northwick Park Hospital; K, King’s College London Guy’s Campus; L, Imperial College; M, Chelsea and Westminster Hospital; N, University of Roehampton London; O, Richmond University; P, Kingston University; Q, St Mary’s University Twickenham London; R, Croydon College; S, Northumbria University, London etc.

The spatial pattern of variable *η*_6_ (figure 2) is associated with universities, which is not expected to be related to ethnicities. However, statistical analysis reveals differences in correlation with various ethnicities. The correlation coefficient between *η*_6_ and the ethnic group of *White: English/Welsh/Scottish/Northern Irish/British* is high at 0.570, while it has a negative correlation with *British only identity*, *self-employed individuals* and the *African language group of Somali*. These correlations are probably due to historical factors, as universities were established at a time when fewer immigrants came to the UK for education, and university communities tend to be selective or stable, with many graduates having a strong emphasis on education and research.

At a finer level of correlation, *η*_6_’s correlation with individuals who identify as having *No religion* is 0.546. This can be explained by the high proportion of non-religious researchers in scientific or social studies, as well as the high proportion of non-religious international students in university-related areas. Other social variables that have correlations with *η*_6_ that are greater than 0.50 include *Born in the UK* (0.530), *Europe: Total* (0.508) and *No British identity* (0.502). Census data were collected at the household level to identify households with pure British or foreign backgrounds in the highly correlated social variables. This household composition is representative of the typical characteristics of university staff and students, including middle-aged families established prior to recent globalization and young students in shared tenancy arrangements.

### 3.3. Social stability: prisons and military installations

Eigenvector *η*_3_ was found to have a high correlation with prison installations, as evidenced by its strong association with the social variable *Other establishment: Prison Service* and *Other establishment: Detention Centers and other detention* (correlation coefficient valued 0.855). This correlation suggests that areas with similar population compositions to prisons are characterized by a unique pattern that may reflect societal instability.

To further validate this association, we examined the correlations of other social variables with *η*_3_. Our analysis revealed that several factors, including race, education and health, contribute to an area’s stability. Specifically, we found that *η*_3_ was positively correlated with *White: English/Welsh/Scottish/Northern Irish/British* (correlation coefficient valued 0.128), *No qualifications* (0.125), *Routine occupations* (0.124), *Fair health* (0.107) and *Last worked before 2001* (0.103).

Of these social variables, health was found to have a particularly interesting relationship with *η*_3_. Our analysis showed that *medium health conditions*, rather than *very good*, *good*, *bad* or *very bad health*, were largely positively related to *η*_3_. This result is intuitive as individuals in perfect health are likely to have adequate income and those in poor health are less likely to commit a crime. Taken together, these findings provide further support for the hypothesis that *η*_3_ is a marker of societal instability, and suggest that the distribution of population characteristics related to race, education and health may play a role in shaping the spatial pattern of crime and prison. These implications are useful for policymakers and researchers seeking to understand and address the root causes of instability in society.

## 4. Feature localization into cities

Our analysis of the 2011 UK Census data revealed substantial city-based heterogeneity among the myriad of social variables. Though these variables hold global importance, they manifest distinct local characteristics that can offer critical insights into city-specific dynamics. It is worth noting that the original definition of OAs from the census was intended to demarcate areas of local homogeneity, designed to be different from their neighbours. However, traditional methods of analysis may not effectively represent the degree of a feature’s localization within a city, especially when high and low values of these features form distinct, non-overlapping spatial clusters. To bridge this gap and illuminate the complexity of social patterns at the city level, we propose the use of the LIPR, as detailed in the Methods section. This approach enables us to more accurately capture the nuances of spatially localized social phenomena. Note that further spatial clustering analyses, employing techniques such as Getis–Ord and local Moran I tests, have been included in the electronic supplementary material.

**Figure 3.**
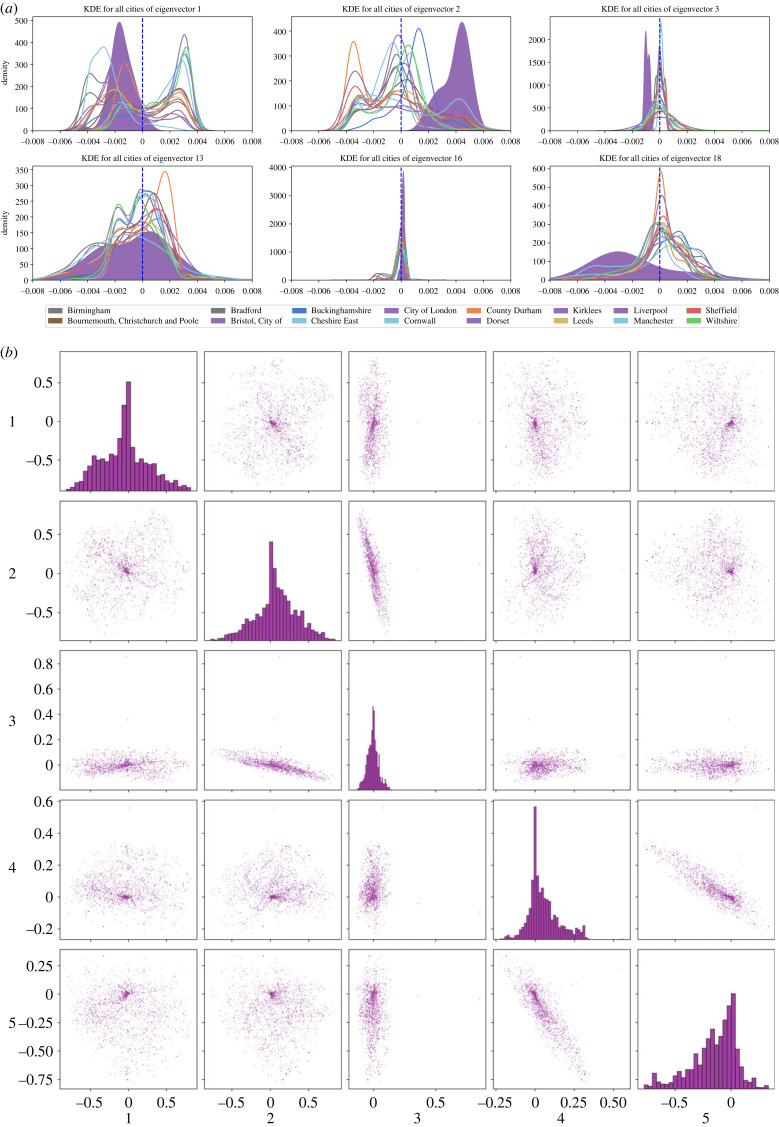
(*a*) Histograms of the values of *η*_1_, *η*_2_, *η*_3_, *η*_13_ and *η*_16_ are grouped by cities, defined according to the 2011 local authorities as listed in the legend. The distribution of *η*_1_ and *η*_3_ values for London are concentrated around zero, indicating that these eigenvectors are not localized for London. Conversely, *η*_2_, *η*_13_ and *η*_16_ display a higher concentration of extreme values in London, resulting in higher LIPR values. These distributions for London are noticeably flatter and broader, highlighting the localization of these eigenfeatures. (*b*) The correlations between the original census social variables and pairs of the five leading eigenvectors are illustrated. Each non-diagonal subplot, indexed by (*i*, *j*), depicts points whose coordinates represent the correlations between a social variable vector and either eigenvector *i* or *j*. This pattern contrasts with the more uniform distribution of correlations in PCA, where each principal component typically represents a broad spectrum of the original variables.

To comprehend the concept and implications of the LIPR more intuitively, we show the aggregated histograms of the entries corresponding to leading eigenvectors *η*_1_, *η*_2_ and *η*_3_, along with selected eigenvectors *η*_13_, *η*_16_ and *η*_18_ in figure 3*a*. These eigenvectors were selected due to their distinct patterns of localization in the cities under study. The entries are categorized by cities according to the 2011 local authorities’ definitions. From the presented data, *η*_1_ and *η*_3_ are interpreted as non-localized eigenvectors for London. This conclusion is based on our LIPR analysis, which reveals a distribution of entries for London that are predominantly centred around zero. In stark contrast, eigenvectors *η*_13_, *η*_16_ and *η*_18_ present high LIPR values for London, suggesting significant localization of these eigenfeatures within the city. This observation is corroborated by the distributions specific to London, which are markedly flatter and broader than those corresponding to other cities. Consequently, this graphical representation provides compelling visual evidence of the utility of LIPR in discerning and comprehending the localization of global factors within distinct urban regions. Figure 3*b* shows the correlations between the original census social variables and the aforementioned eigenvectors. Each non-diagonal subplot represents the correlation between a social variable vector and a particular eigenvector *i* or *j*. A unique pattern, distinct from the homogeneous correlations seen in PCA, emerges. The relatively limited number of social variables demonstrating strong correlation with eigenvector *η*_3_ further underscores the value of our LIPR analysis for comprehending the multifaceted relationships between eigenvectors and social variables.

We investigate London to show how the LIPR is used. First we determine the study set of the first 20 eigenvectors, to pinpoint some of the properties that are important aggregation of social variables valid for the whole EW. Then for each of the eigenvectors, we query the entries that correspond to the OAs in London and further compute the LIPR for the eigenvector–city pair (table 2). A benchmark for LIPR^London^ values is the uniform distribution, where a feature takes the same value of $1/\sqrt{N}$ in all the OAs in EW, where *N* = 181 408. In the Greater London region, there are *N*_London_ = 24 927 OAs, and the corresponding ‘neutral’ LIPR value is LIPR* = 24 927/181 408 = 0.137408. For an eigenvector *η*_*i*_, if its ${\mathrm{LIPR}}_{i}^{\text{London}}$ is greater than LIPR*, it can be referred to as a *localized* feature in London; otherwise, if ${\mathrm{LIPR}}_{i}^{\text{London}}$ is smaller than LIPR*, *η*_*i*_ is not a localized feature in London (either not localized at all, or localized in other cities). A localized feature in London refers to a unique and distinguishable community that is highly concentrated within the city of London, setting it apart from its surrounding neighbourhoods. Specifically, if an eigenvector has a high inverse participation ratio (IPR) but a low LIPR, it means the corresponding feature is globally significant but not localized in the city. On the other hand, if a feature (such as prisons) has a high IPR and a low LIPR of a city, the feature usually corresponds to those rarely seen but essential elements for every city thus infrastructures.

**Table 2.** The ranked LIPRs of eigenvectors restricted in London. The larger LIPR of an *η* indicates that the feature is more localized in London. Generally, the features with small LIPRs are infrastructures, while the features with larger LIPRs are the superlinear urban indicators.

indices of *η*	LIPR	interpretation
3	0.010373	prison service
8	0.011114	educational establishment
4	0.022512	defence establishment
9	0.026599	retirement
10	0.030878	defence
7	0.058357	full-time employee
5	0.064503	one person household/household spaces with no usual residents (tourist)
15	0.066020	multi-person household: all full-time students averaged household spaces
6	0.077067	University
0	0.138103	—
12	0.143874	one car or van in household/lower supervisory and technical occupations
14	0.179744	owned: owned with a mortgage or loan/economically active: employee: full-time
19	0.261445	one family only: married or same-sex civil partnership couple: all children non-dependent/other households: three or more adults and no children
17	0.264369	intermediate occupations/multiple types of central heating
1	0.274687	marriage/many vehicles/redundant rooms
11	0.295386	skilled trades occupations/lower supervisory and technical occupations/caring, leisure and other service occupations
2	0.303946	higher degree (finance and technology)
16	0.567684	Tamil/opposite: Yiddish/Israeli
18	0.716807	gas central heating/solid fuel
13	0.743838	gas central heating, three or more adults and no children, highest level of education

The LIPR-ranking approach allows for a systematic investigation of the small social groups in the country who gather in London and have a significant impact. The top localized features in London are LIPR_13_ = 0.74, LIPR_18_ = 0.72, LIPR_16_ = 0.56, LIPR_2_ = 0.30, LIPR_11_ = 0.29, LIPR_1_ = 0.27, LIPR_17_ = 0.26, LIPR_19_ = 0.26, LIPR_14_ = 0.18 and LIPR_12_ = 0.14 (in descending order). The rest of the eigenvectors may not be localized in London, but could be localized in other cities.

**Figure 4.**
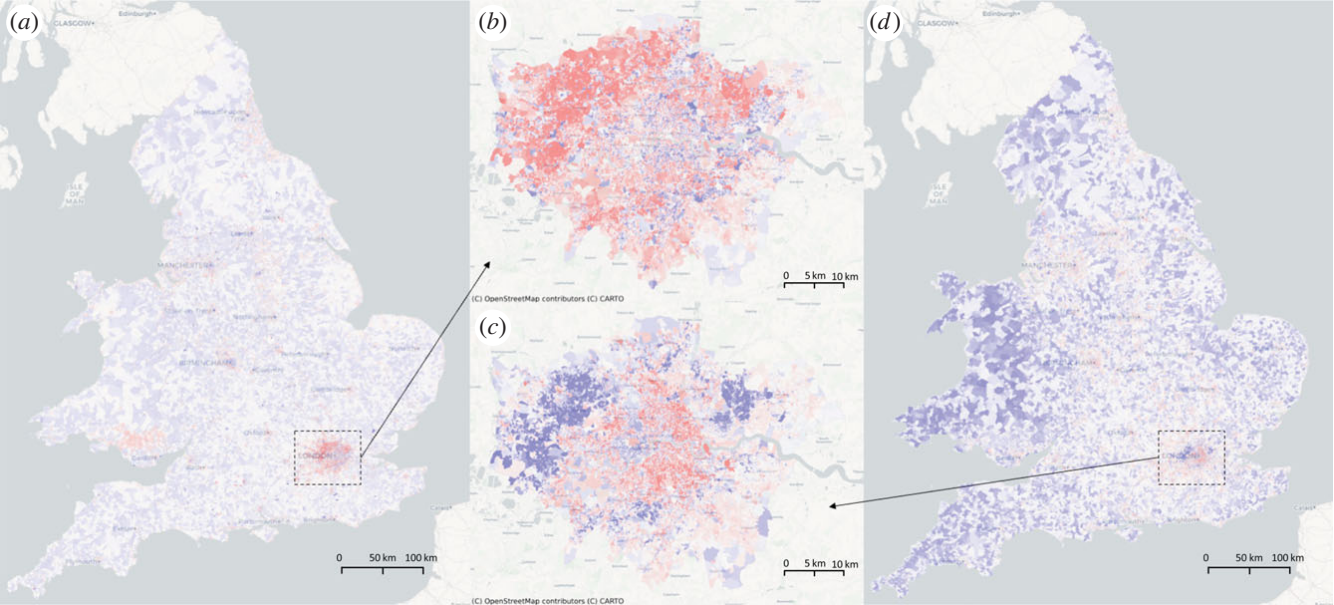
The most localized features in London, *η*_13_ and *η*_18_ for typical lifestyles. Here, (*a*) and (*b*) are the spatial map of *η*_13_ for England and Wales, and London, respectively; (*d*) and (*c*) are the spatial map of *η*_18_ for England and Wales, and London, respectively.

The correlation analysis of localized features in London reveals that *η*_13_ and *η*_18_ (figure 4) are highly associated with central gas heating, highest level of education and households consisting of three or more adults with no children. The correlation coefficients of these features with *η*_13_ and *η*_18_ are around 0.25, which highlights the demographic composition of the typical Londoner. Central heating is more prevalent in newer and more expensive homes, and these homes are more likely to be occupied by higher-educated and childless individuals. The concentration of such households in the affluent suburbs of London is consistent with the trend of urban gentrification and high demand for modern and comfortable living environments in urban areas. Our findings suggest that this demographic is characterized by well-educated individuals living in new build properties with central gas heating. The spatial distribution of *η*_13_ highlights the affluent suburbs of London, which suggests that this area is perceived as desirable by wealthy families in business. This finding is supported by previous research studies [25], which have demonstrated a positive relationship between education level, household composition, and central heating system with wealth and urban development.

**Figure 5.**
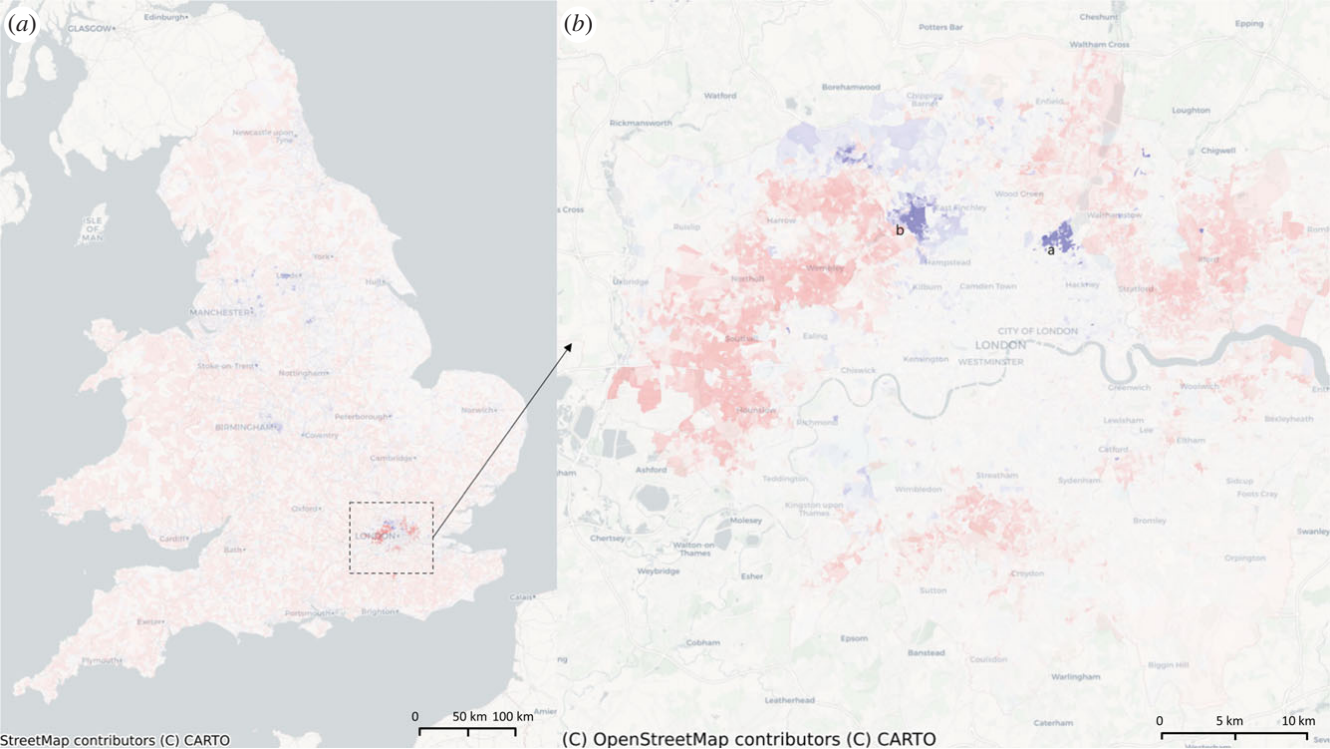
The spatial clustering of Tamil-related people found by *η*_16_ of England and Wales (*a*) and London (*b*). Here, (*a*) is Tamil Community Housing Association, and (*b*) is Tamil Association of Brent. The darkest red regions are however the clusters of Yiddish and Hebrew speakers.

The third highest localized feature in London, *η*_16_, is associated with social variables related to the Tamil community and Yiddish, Israeli and Hebrew speakers, as shown in figure 5. Negative entries of *η*_16_ indicate the presence of the Tamil community near the Tamil Community Housing Association, which supports refugees from Sri Lanka. The Tamil community in London has been growing since the Sri Lankan Civil War and is becoming distinct, as evidenced by high academic performance of Tamil children and a preference for having only children. Meanwhile, positive entries of *η*_16_ mark areas with high concentrations of Yiddish, Israeli and Hebrew speakers in Stamford Hill, North London. These areas tend to be isolated, as seen in the distribution of Yiddish newspapers aimed at audiences in Leeds, Manchester and Gateshead, rather than being clustered in a distinct Yiddish neighbourhood.

The two eigenvectors, *η*_2_ and *η*_11_, provide insight into the occupational landscape of London. *η*_2_ is highly positively correlated with areas that demand degrees and higher education, such as BA, BSc, MA, PhD and PGCE, with a correlation coefficient of 0.85. This indicates a strong presence of professional and highly educated individuals in these areas. On the other hand, *η*_11_ marks communities with a higher concentration of lower supervisory and technical occupations, including mechanics, chefs, train drivers, plumbers and electricians, with a correlation coefficient of 0.33. These are typically considered higher grade blue-collar jobs that require specialized skills.

It is worth noting that *η*_2_ also has a negative correlation with South Asian language speakers, specifically those who speak Pakistani Pahari, Mirpuri and Potwari, indicating a lack of assimilation into London’s societies. This may suggest a potential barrier for these individuals in accessing higher education and professional opportunities.

**Figure 6.**
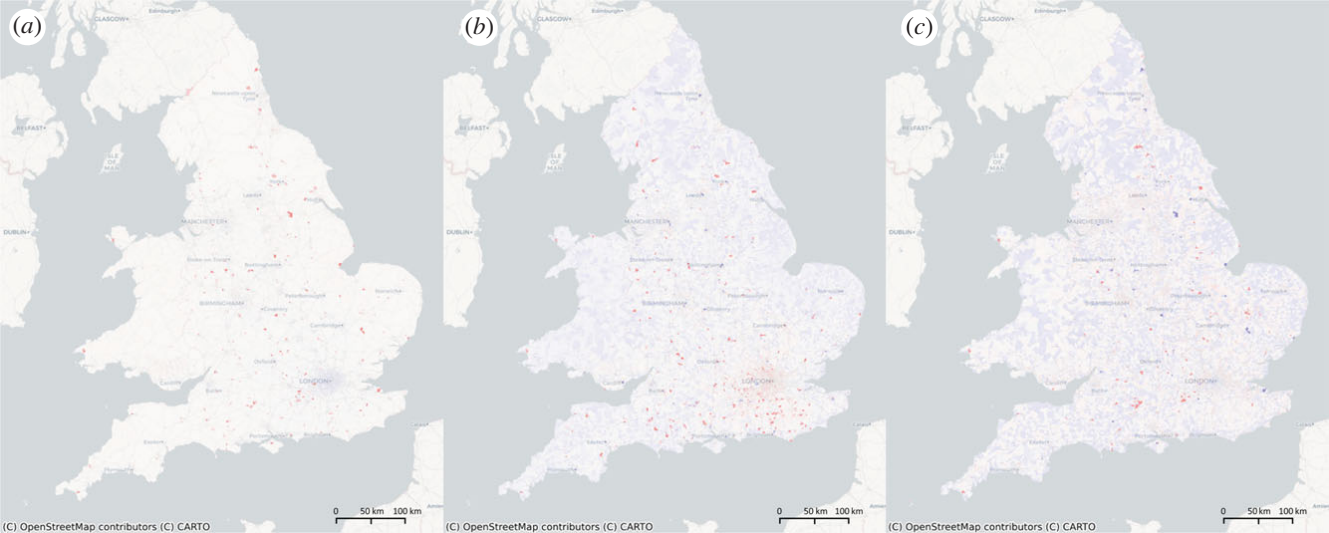
Three of the most globalized eigenvectors: (*a*) *η*_3_ maps prison installations; (*b*) *η*_8_ maps educations before college; and (*c*) *η*_4_ highlights the military camps.

The eigenvectors identified by *η*_3_, which highlight prison installations (figure 6*a*), are not unique to London, but can also be found in other cities. This eigenvector has the highest level of non-localization among the first 20 (with a correlation coefficient of 0.010373), indicating that these features may be associated with broader infrastructure elements, such as *η*_8_ for education (figure 6*b*) or *η*_4_ for national defence (figure 6*c*). This highlights the importance of considering the broader contextual factors that influence local patterns and structures in cities, beyond just their specific local features.

## 5. Discussion

In this article, we applied DMs to analyse the synchronized variations in the census responses of EW. Our study represents a novel attempt to decompose the British census as a whole, not just in urban areas. The results of our work demonstrate the effectiveness of DMs in uncovering the underlying social structures in bulk, publicly accessible data. Our method ranks the relative importance of different features by themes and highlights the continuity of social aspects, such as educational levels, in the form of continuous indices.

The complex nature of demographic features calls for an efficient and scalable data analysis approach that can handle multiple scales and themes. Manifold learning methods, such as DMs, are ideal for this purpose as they focus on local structures while preserving global information. To adapt the DMs method to the bulk census data of EW, we developed several techniques, including the preservation of a limited number of correlations that ensure connectedness and the use of Spearman Rank Correlation to measure the high-dimensional census data and account for heterogeneity in the distribution of social variables.

The diffusion mapping eigenvectors shed light on the urban structures of EW and their impact on the cross-scaled behaviours of British society. For example, at a global level, the DM reveals general patterns of social deprivation and the spatial distribution of various social variables across the whole of EW led by urbanization from *η*_1_. At a local level, our method identifies specific clues of connected urban areas of walkable neighbourhood in *η*_1_. Further, we identified small-scale hotspots of inequality, small-scale characteristics of a feature localized in big cities, and spatial clustering of small communities. These local insights contribute to a detailed understanding of demographic variations, thus capturing both the broad strokes and fine details of societal structure.

Our method uses a heuristic definition of the *k* nearest neighbour network to ensure that these characteristics are globally sensible and applicable to all areas, not just cities. Furthermore, the LIPR is used to discern features as sublinear or superlinear urban indicators using only one input dataset. Therefore, our approach can manage large cross-scale problems by revealing both the general picture and local nuances within one unified model.

The proposed LIPR is a method for identifying and characterizing small-scale characteristics of a feature (such as minority groups, prison establishments etc.) in urban areas. The LIPR measures the concentration of a given feature in a specific region compared with its distribution across the entire urban area. It calculates the fraction of total variation in a feature that is captured by a limited number of OAs. The LIPR values of each feature allow us to categorize the features as either sublinear or superlinear urban indicators. Features with high LIPR values are considered highly concentrated in one region and classified as superlinear indicators, while features with low LIPR values are considered widely distributed and classified as sublinear indicators.

Our findings extend the existing knowledge that some urban indicators, such as the number of university students, can be infrastructural in some cities but are urban outputs in the others. The LIPR provides valuable insights into the distributional patterns of demographic features in urban areas and can reveal the unique social, economic and cultural characteristics of highly concentrated minority groups and their relationship to the broader urban population.

## Data Availability

The 2011 census data of England and Wales can be retrieved from Office for National Statistics of the United Kingdom: https://www.ons.gov.uk/census. We downloaded a bulk version from NOMIS: https://www.nomisweb.co.uk/census/2011/bulk/r2_2. The data are provided in electronic supplementary material [26].
